# Love and Human Rights

**DOI:** 10.1093/ojls/gqac034

**Published:** 2023-01-11

**Authors:** Benedict Douglas

**Affiliations:** Durham University

**Keywords:** human rights, family law, immigration law, love, sexuality, legal theory

## Abstract

This article explains and critiques the protection of love within judgments concerning relationships under the Human Rights Act 1998. Using theory of emotion to conduct doctrinal analysis of the protection of love within international human rights laws and under the Human Rights Act 1998, it reveals a shift in the conception of love underlying the domestic judicial application of huamn rights. Whereas previously the law was underpinned by values of duty and property, judgments concerning relationships now protect the capacity of individuals to choose how to live. However, the protection of this modern conception of love is limited by judicial deference, allowing the values underpinning the historical conception of love to continue to influence the law.

## 1. Introduction

The constancy of love as a theme in music and literature shows its importance to us. Human rights recognise and protect our equal inherent dignity, and specific rights recognise the importance of our relationships as part of this.^1^ However, love has different meanings, and rights that protect relationships do not explicitly mention or define love. Therefore, we need to ask what protection is and should be given to love in the interpretation and application of human rights. This article focuses on the UK to assess the extent to which the Human Rights Act 1998 (HRA 1998) has led to a change in the conception of love recognised and protected by the courts in cases concerning relationships. I argue the incorporation of human rights has changed the conception of love within our law to a more modern conception, one consistent with the moral basis of human rights in the capacity of individuals to choose how to live.

I begin by defining love using Martha Nussbaum’s argument that different accounts of love are distinguished by the moral judgments they contain.^2^ Supporting and critiquing her analysis using the work of psychologists John Bowlby and Erich Fromm, and further literary analysis, I argue the modern account of love shares a moral foundation with human rights, including the European Convention on Human Rights (ECHR, the Convention), in the capacity of individuals to choose how to live. In the subsequent section, I demonstrate that the conception of love implicit within law concerning relationships prior to the HRA 1998 was one predominantly defined by the values of obedience to duty and property, reflecting their historical influence within our society and law.^3^ Finally, I argue that, under the influence of the HRA 1998 and the value of individual choice underpinning the ECHR, the courts have led a departure from the historical understanding of love to substantially recognise and protect the modern conception of love.

But this shift is not yet complete. It has been limited by judicial deference, particularly in the context of immigration. I argue this shows the values of duty and property underpinning the historic conception of love still have force within our law and society. The HRA 1998 has caused significant change in the values protected by the courts, but we still have some distance to travel to respect and protect the conception of love consistent with the moral basis of human rights.

## 2. What Is Love?

Through arts and sciences people have sought to understand love and its place in human experience and action.^4^ So much so that it is not possible to look at every account of love, but two elements are common to different highly influential accounts of it. Love is a desire for togetherness with another, which affirms the identity of both the lover and the beloved, the values of the lover and value of the beloved.

Love is difficult to define because we each love different people and the feeling of desire for togetherness with another recognised by societies as love has changed over time. This is because emotions are not simply feelings produced by biological states within the body. Emotions involve an individual’s judgment about an object or state of affairs based on their experiences, which gives them their values, composed of their moral, aesthetic and other opinions.^5^ For example, feeling fearful walking down a dark street involves a judgment informed by the value of one’s life, past experience and knowledge about the dangerousness of such situations. This judgment generates the mental state of fear and bodily preparation for fight or flight. It is similarly different judgments of value that give rise to the different definitions of love and cause us to love different people.

Whether we love a particular person, and the nature of the desire society recognises as love, depends on what a person or society judges to be worthy of a desire for togetherness based on their values. Different types of love were described by the ancient Greeks—*eros*, *agápe*, *storge* and *philia* (desire, charity, affection and friendship)—because of the different things that are valued.^6^ At an individual level, what can be valued in another can be their beauty, power or property if that is valued, or anything else about the beloved that is judged as affirming the lover’s values.^7^ It can be obedience to duties if this is what the lover values. It can be their freedom of choice or their particular choices that give them their identity. We love different people because we value different things. We can love things other than people—animals,^8^ objects,^9^ places,^10^ countries,^11^ ideas—if the value we give to them leads us to desire to be with them or identified with them, and affirms their identity and our own. At a societal level, we have had different accounts of what love is because, throughout history, the influential definitions of love within Western society have reflected the prevailing moral values of the time.^12^

### A. Desire for Togetherness

The desire for togetherness with another was present in Plato’s early account of love.^13^ In his myth, humans were once spherical beings of three genders, with four hands and legs, and two faces, who were divided into two by Zeus after attacking the gods.^14^ Consequentially, we feel compelled to wander looking for our other half, seeking to unite again to achieve the wholeness of our ‘original nature’.^15^ Although conflicting with modern understanding of human evolution, its metaphor contains the idea of a desire for togetherness as a core element of love.^16^

Psychology recognises a desire for togetherness with others as fundamental to human beings. John Bowlby, in his ground-breaking work on attachment, describes it as one of our earliest instincts: ‘No form of behaviour is accompanied by stronger feeling than is attachment … the figures towards whom it is directed are loved.’^17^ Interfering with this has a fundamental impact upon an individual’s psychology.^18^ Focusing on adults, Erich Fromm, in his influential *The Art of Loving*, draws on psychology to describe how an individual’s ‘awareness of his aloneness and separateness, of his helplessness before the forces of nature and society … makes his separate, disunited existence’ unbearable.^19^ He thus argues that ‘[t]he deepest need of man … is the need to overcome his separateness’: love is the means by which separateness is overcome.^20^

As part of Western legal culture, English ‘law tends to reflect … an individualised understanding of the self’.^21^ It focuses on individuals, their obligations and rights in relation to others.^22^ In contrast, Asian legal culture’s collectivist approach subordinates the individual to the community, prioritising duties towards society and others, and African legal culture’s focus is on the person as a member of the community and ensuring peace within the community, for example the *ubuntu* value.^23^ Nonetheless, consistent with the fundamental nature of the desire for togetherness, English law has long given recognition to relationships.^24^

Since the Universal Declaration of Human Rights 1948, international human rights laws have similarly acknowledged the social nature of humans and the desire for togetherness with others that is part of love, recognising the importance of relationships ‘as the foundation of both society and the state’.^25^ Some rights explicitly protect family relationships and freedom of association, but so important is togetherness that even more individual focused rights protect it. Under article 3 ECHR, solitary confinement can amount to inhumane treatment because it can ‘ultimately destroy the personality’.^26^ The importance of relationships is explicit in the article 12 ECHR right to marry and found a family. Although this right has been interpreted narrowly, as covering only marriages between different-sex couples and conferring no rights on unmarried couples or children,^27^ article 8’s protection of ‘family life’ has been expansively interpreted. The European Court of Human Rights (ECtHR) has not interpreted it as merely requiring respect for relationships already recognised by law, the interpretation preferred by British judge Sir Gerald Fitzmaurice,^28^ but as imposing an open-ended positive obligation on Member States to recognise ‘other *de facto* “family” ties’.^29^ What amounts to family life is decided on the facts of each case.^30^ Its interpretation has developed to include same-sex relationships,^31^ and the Court has become less willing to grant a margin of appreciation on its application, recognising that a ‘particularly important facet of an individual’s existence or identity is at stake’.^32^

As with the ECHR, under UN treaties relationships are protected within an ‘extremely wide understanding of “family”’.^33^ Article 23 of the International Covenants on Civil and Political Rights and article 10 of the International Covenant on Economic, Social and Cultural Rights describe the family as the ‘natural and fundamental group unit of society’, and article 10 further states family life should therefore be accorded the ‘widest possible protection and assistance’.

Only the UN Convention on the Rights of the Child recognises love explicitly, but not as a right, for reasons I will explain shortly.^34^ Its preamble states that ‘for the full and harmonious development of his or her personality, [a child] should grow up in a family environment, in an atmosphere of happiness, love and understanding’.

### B. Affirmation of Value

The desire for togetherness is a constant throughout the history of thought on love.^35^ However, it is the nature of the desire that makes a desire for togetherness love, as opposed to possession or ownership, and distinguishes different conceptions of love. Love is a desire for togetherness that affirms the value of the lover and the beloved. At an individual level, what it is about the other that is valued and affirmed reflects the individual’s own experience and values, which are in turn affirmed within the relationship.^36^ What is recognised within society as love is determined by the predominant moral values of the time.

Thus what is recognised in each era as love is a moral judgment.^37^ Nussbaum conducts a ‘enormously ambitious’ survey of the Western literature on love of different moral eras to argue that accounts of love have changed in a way that reflects stoic, religious, romantic and modern values, changing with the morality of the time.^38^ English law’s recognition and protection of love similarly reflects the changing predominant moral values of our society.

Plato’s description of love is consistent with stoic morality. For him, the other that we should desire is not a single particular person. Rather he argues that love is the desire for the ultimate good, the beautiful, which is a state of being in which an individual continually seeks knowledge of the true nature of any and every person or object.^39^ I think John Keats expresses this in his *Ode on a Grecian Urn*, ‘beauty is truth, truth beauty—that is all/ Ye know on earth, and all ye need to know’.^40^ Plato argues that the desire to know and affirm the nature of all persons and objects will enable an individual to be flourishing and complete, a good person.^41^

Nussbaum argues that the Christian religious morality that subsequently predominated in the West contained an idea of love grounded in the value of dutiful obedience to God. St Augustine of Hippo, the 5th-century North African theologian, put forward an account of love reflecting this religious morality. He argued that love for other individuals should be guided by the love of God, as the proper focus of the individual’s life should be upon obedience to God’s will.^42^ In his *Divine Comedy*, Nussbaum argues that Dante shows the grounding of a Christian idea of love in duty.^43^ In his guided tour through the underworld, which Vittorio Montemaggi conversely argues we should read as a criticism of this notion of love, only people who obey the religious rules are worthy of divine love; others are condemned to the relevant circle of hell.^44^

As the influence of duty based religious morality waned during the Enlightenment,^45^ so ideas of what should be loved about the other also changed. Nussbaum argues that in *Wuthering Heights* Emily Brontë rejects what she perceived to be the Christian account of love in her time.^46^ Cathy’s dream, in which ‘heaven did not seem to be my home … and the angels were so angry that they flung me out’ sobbing with joy to return to Wuthering Heights, embodies Brontë’s criticism of a Christian love that denies the individual’s freedom to choose.^47^ Heathcliff embodies Brontë’s preferred form of love: his love is a free expression of his own will unconstrained by the morality of the time, and his affirmation of Cathy’s freedom of choice is seen in his acceptance of her decision to marry Linton.^48^ Conversely, Cathy tragically feels unable to choose to be with Heathcliff because his alien-ness to her society and class conflicts with the duties both impose upon her.^49^

In *Jane Eyre*, by Emily’s sister Charlotte, we see a similar shift to an idea of love valuing the individual as a choosing being, away from Christian love defined in terms of duty.^50^ The justification for Jane’s rejection of the proposals that she become Mr Rochester’s mistress or the Rev St John’s missionary wife is her view that to be either would be to be untrue to her own values and identity. She rejects the former to ‘hold to the principles received by me when I was sane, and not mad—as I am now’, and the latter so that her ‘heart and mind would be free’.^51^

Consistent with the foundation of 20th-century rights based morality and each other, Nussbaum’s literary criticism and Fromm’s psychology put forward an idea of love grounded in the conception of the individual as a being able to choose how to live and defining their individual identities through their choices. Under their theories, the desire for togetherness affirms the unique individual identity of both the lover and the beloved as beings capable of freely choosing how to live.^52^ Although the literature of the Romantic period also values individual choice, Nussbaum argues that it gives an unrealistic account of love because the beloved is idealised beyond reality.^53^ The romantic account does not fully recognise and affirm the individual as real persons, separate and choosing. This is more true of Emily than Charlotte, whose account of her characters is more detailed and modern.

Fromm, critiquing duty based Christianity and the focus of capitalism on individual wealth in Western society,^54^ describes love as a paradox uniting an individual with others and yet preserving the integrity of the individual identities of the lover and the beloved.^55^ This he distinguishes from accounts of love premised on the obedience of the individual to the beloved or on the pursuit of happiness of separate individuals.^56^ Togetherness is, he argues, possible under forms of love characterised by dutiful obedience or individual exchange. In the former it involves, like patriarchal religions under which God’s love is conditional on obedience,^57^ a submission of the will of one to another that does not respect the capacity for choice of whoever submits,^58^ and in the latter a mutual exchange of personalities in the hope of individual benefit.^59^ In contrast, the love Fromm advocates is founded in recognition of the common nature of humans as individuals capable of choosing how to live.^60^ Thus, he argues that to love another is to enhance another’s sense of aliveness in a way that also enhances one’s own;^61^ to love is both to manifest one’s own and to affirm another’s human capacity for identity defining freedom of choice.^62^

Nussbaum argues that this modern account of love is most fully embodied in James Joyce’s *Ulysses*, underlying the political stance within it.^63^ For Joyce, love involves the recognition of an individual’s whole being, their entire flawed humanity,^64^ and compassion in recognising their needs, desires and choices.^65^ A desire for togetherness is present in the protagonist Bloom’s rejection of discrimination and argument that common humanity unites all people,^66^ and his relationship with Molly where, knowing of her infidelity, his anger is overtaken by his love for her, his desire to remain with her.^67^ The affirmation of the identity of the beloved as a choosing being is present in Joyce’s recognition that love involves embracing the fact that the beloved is beyond the individual’s control, the inconsistency and imperfection of real people.^68^ By not being required to desire a particular type of person, an individual is free to love wisdom, God or another, so long as in doing so they do not cease to love their whole self and that of others, the choices and desires that characterise their lives.^69^ Written during the coming of Irish independence, Joyce’s account of love critiques the duty based conservativism of those who wished to maintain the coercive control of the British state and morality of the ‘Catholic lower class’.^70^

Human rights are similarly grounded in and protect the capacity of the individual to choose how to live and, through so choosing, to define their identity. Ronald Dworkin argued there is a widespread view of the equal intrinsic value for individuals of their own lives and choosing for themselves how to live, and in so doing create their own meaning or narrative for their lives.^71^ He argues these two principles of human dignity form the fundamental justification for human rights.^72^ Similarly, Nusbaum and Amartya Sen argue human rights are grounded in and protect the ability to choose how to live.^73^ The personhood that James Griffin argues human rights are grounded in, the dignity that UN human rights documents protect, is the capacity to choose how to live, and the rights protect the liberty to do so.^74^ Looking in more detail at rights documents, including the ECHR, Kai Möller argues that they are grounded in and protect the autonomy of individuals, their control of their body and their choice of how to live through which they develop their self-conception of their own identity.^75^ Although there are differences in the theories they put forward,^76^ they agree on the capacity to choose how to live as foundational for human rights.

Consistent with this analysis of rights documents, the ECtHR in *Pretty v UK* recognised that ‘self-determination runs like a thread through the Convention’,^77^ protecting individual autonomy, the capacity ‘to conduct one’s life in a manner of one’s own choosing’.^78^ Thus, the affirmation of the individual identity of both the beloved and the lover as beings with the capacity to choose how to live, which is the substantive content of Nussbaum and Fromm’s account of the modern conception of love, is supported by the value underlying human rights and the Convention.^79^

Although on its face this basis appears individualistic, human rights do not view humans as solipsistic beings. The recognition of rights is a recognition that humans live as part of a community with different interests and desires. Human rights attempt to balance conflicting choices in a way that protects the individual against being obliterated by the interests of others with greater power.^80^ They do not merely exist to prevent the rise of an undemocratic or despotic government;^81^ they are a means by which to challenge the exercise of power and balancing of interests within a state. Most rights are stated in a qualified manner, subject to exceptions, recognising an individual’s rights may be limited in favour of other’s. Consistent with human rights, the modern conception of love does not mean absolute unlimited freedom of choice for any one individual; it draws individuals together whilst preserving the capacity for choice of the lover and beloved.^82^

Although rights treaties recognise and protect relationships, none state a right to be loved. Such a claim that another has a duty to love a particular individual would be inconsistent with a basis of human rights in the ability of an individual to choose how to live.^83^ The lack of a right to a divorce within the ECHR is inconsistent with this; it is a deliberate omission to make the Convention acceptable to countries strongly influenced by a Catholic duty based conception of love prohibiting divorce.^84^ Despite this, the ECtHR has protected a choice based understanding of relationships. In *Airey v Ireland*, the ECtHR held that whilst there was no right to divorce, article 8 ECHR protected a right of married persons to be ‘relieved from the duty to live together’ where one chooses to end the relationship.^85^ More recently, the ECtHR has diminished the margin of appreciation, finding that where national law allows divorce, procedural obstacles may violate the Convention.^86^ However, the margin of discretion remains ‘wide’,^87^ and recently dissenting judgments strongly argued this was inconsistent with the importance of ‘personal choice regarding marriage’.^88^

This protection of individual choice within relationships can also be seen in the extent and limits of what is protected as family life under the ECHR and other rights treaties. As well as requiring the recognition of relationships, the rights documents have been interpreted as requiring states to facilitate the material conditions for family life, protection from violence and discrimination.^89^ Fareda Banda and John Eekelaar argue that the protection given to individuals against discrimination and violence within relationships,^90^ and the observations of treaty bodies against polygyny, levirate marriage and marriage following rape,^91^ constitute normative limits on what will be protected as family life.^92^ This is consistent with a conception of love that protects individuals’ equal value in their capacity of choice in relationships.

Love is an emotional desire for togetherness, an attitude to another that reflects human evolution in the fact that we love, and our own personal history and values in terms of who we love. The desire recognised by society as love has changed throughout the course of human history, in a way that reflects the prevailing moral values of the time. Human rights, grounded in the capacity of individuals to choose how to live, recognise and protect us as choosing and relational beings, supporting the modern conception of love. I will argue below that the HRA 1998 has led to increased protection of the modern idea of love in the UK by the courts as a consequence of the influence of the moral value of individual choice underpinning human rights, departing from the previous focus on obligations in relationships. Each generation may not invent sex, but each era redefines love.

## 3. The Place of Love in the Law prior to the Human Rights Act

Given the importance of love to human beings, and its reflection of the morality of each age, it is unsurprising that the modern account of love is consistent with the values underpinning human rights. However, the UK has only recently legislated to recognise human rights, and in this section I draw together arguments to show that, prior to the HRA 1998, the law recognising and regulating relationships was predominantly shaped by the values of duty and property.

John Eekelaar argues that historically English law concerning family relationships has been strongly characterised by duties.^93^ Marriage was ‘directed primarily at securing adherence, as far as possible, to a particular social order’,^94^ it was viewed as a contract creating an institution of ‘financial [and] procreative [security], and social standing’.^95^ The effect of these duty and property values has influenced the recognition and protection of love in substantive laws and judicial interpretation into the 21st century.

Although consent by the parties to a marriage has been required in English law since the 12th century,^96^ and parental consent has not been a general prerequisite, deeper analysis shows this is not indicative of a choice based conception of love. The Clandestine Marriages Act 1753 (26 Geo II c 33), which required parental consent for marriages of those under 21, although short lived and of limited effect on anyone except the wealthy, evidences wider social control.^97^ It reflects a prevailing morality, alongside social and economic pressure, that made it ‘the duty of a child to attempt to obtain’ parental consent.^98^

Within the law on marriage, the duty conception of love underpinned the doctrine of legal unity, the duty to live together and fault-based divorce. The doctrine of unity, grounded in a religious idea that wives should be subservient to husbands, fused husband and wife into one legal person.^99^ Under this the man had control and the wife lacked legal personhood, including the ability to own property. This was diminished by the Married Woman’s Property Act 1870, but not abolished until *Midland Bank Trust Co Ltd v Green (No 3)* in 1982.^100^

Flowing from the same conception of relationships was the doctrine of consortium, an ‘enforceable duty of husband and wife to live together’.^101^ This duty of the wife to submit and care for the husband and of the husband to protect and support her underpinned several causes of action.^102^ Whilst similarly diminished through legal change, it was only declared defunct in 2010.^103^

The requirement to prove fault in divorce was only legislatively repealed in 2020 following a court challenge.^104^ This recent change shows the persistence of a focus on the fulfilment or breach of obligations as central to the existence and end of a marriage into the 21st century.^105^

In private law more generally, the contract law presumption against an intention to create legal relations between family members shows a lack of protection for choices made within relationships.^106^ In negligence, the duty of care at the core of liability was explicitly founded on a moral duty to love one’s neighbour.^107^ Meanwhile, in liability for causing psychiatric harm, the courts have been reluctant to find sufficient proximity for friends or siblings, whose relationships are not characterised by the legal and moral duties owed by parents and spouses.^108^

His study of family law leads Eekelaar to conclude there has been a ‘lack of concern for love in marriage’.^109^ However, love was not absent from this part of the law, or other elements of the law that are beyond the scope of this single article,^110^ but rather here it predominantly reflected a duty conception of love. Although not analysing different conceptions of love, Eekelaar does implicitly recognise this, noting love can be seen as ‘the product of the practice of a duty’ and marriage law involved duties that were not easily dispensed with.^111^

The influence of the value of duty in family law is consistent with the wider influence of duty within our society and legal system. Walter Bagehot argued UK society has historically been underpinned by values of duty: ‘our constitution is not based on equality … but upon certain ancient feelings of deference’.^112^ Historical sources support this characterisation,^113^ and current quantitative research shows obedience to authority and the law (even if wrong) are still predominant values within our society.^114^

In our constitution and private law the long-standing dominance of the value of duty is apparent. As parliamentary sovereignty continues to be the or a ‘fundamental rule’ of the UK constitution^115^—stating that the ultimate duty of all is to obey the will of Parliament—duty continues to be the foundation of our constitution.^116^ Similarly, the ‘core’ of the rule of law, although tempered slightly by judicial development of the presumption of liberty, the principle of legality and the statements in *R (Jackson) v Attorney General*,^117^ states ultimately that all have an obligation to obey the law.^118^ In private law, a considerable proportion that is not concerned with property is called the law of obligations because of its core concern with a failure to perform one’s moral duties, to which rights are corollaries.^119^

The HRA 1998 was intended to alter this, to ‘modernise our society and refresh our democracy’, to change it into a culture of rights.^120^ The moral dimension of this culture is one in which individuals possess ‘rights as an affirmation of their equal dignity and worth’.^121^ In the context of the law governing relationships, it has to a significant extent succeeded.

## 4. Love in the Time of Human Rights

The effect of the duty and property conception of love has significantly diminished as a consequence of the creation of the HRA 1998. In this section I argue that, influenced by the ECtHR’s grounding of the Convention in the individual’s capacity for choice, the courts have given recognition and protection to a conception of love underpinned by the ability of the individual to choose how to live and who to love. That this is a consequence of the HRA 1998 is apparent from both the substance of the judgments and the way in which the courts’ decisions have preceded the recent major legislative changes, recognising and protecting choice in relationships.^122^

However, despite the increased protection of the modern conception of love in the last 20 years, I will argue the values of duty and property remain powerful within our society, limiting the protection of the modern conception of love in some cases. Even with the powers of sections 3 and 4 HRA 1998, which have enabled the courts to challenge the historical conception of love, concerns of constitutional competence have limited the protection the courts can give against these values. In *R (MM (Lebanon)) v Secretary of State for the Home Department*, the protection of the modern conception of love reached its limit.^123^ The courts have been able to protect relationships against the effect of immigration laws in individual cases. But when an immigration rule was directly challenged in *MM*, the court found itself compelled to show deference to the rule that valued relationships in terms of duty and property, confirming the continuing presence and influence of the historic conception of love within our society and law.

### A. Changing Recognition of Relationships

*Ghaidan v Godin-Mendoza* was the first HRA 1998 case requiring judicial engagement with love. It concerned the statutory provision that the spouse of a protected tenant or person ‘living with the[m] … as his or her wife or husband’ could succeed to the tenancy upon the tenant’s death.^124^ The question was whether this included partners in same-sex relationships and, if not, was it in violation of articles 8 and 14 ECHR?

In finding a violation, Lord Nicholls and Baroness Hale described the nature of relationships protected by article 8. Lord Nicholls, giving the leading judgment, argued both different- and same-sex couples ‘share each other’s life and make their home together’, and thus there is ‘no rational or fair ground for distinguishing the[m]’.^125^ For him, the togetherness of a shared life and shared identity was the key feature of the relationships to which the Rent Act 1977 was intended to apply,^126^ and used section 3 HRA 1998 to include same-sex couples.

Baroness Hale engaged directly with the emotional aspect of relationships, describing love as involving ‘not only the warmth but also the sense of belonging to one another which is the essence of being a couple’.^127^ Read alongside her reliance on the ECtHR’s statement in *Pretty v UK* of the Convention’s basis in the protection of individual choice,^128^ she recognised and applied the modern conception of love grounded in the mutual affirmation of the individual identities of the partners shaped through their own choices. The majority’s decision gave legal recognition to people’s choice to enter a relationship with a person of either sex. At a deeper level, it was a significant departure from the law’s historical approach to relationships which reinforced the duties to maintain the social order through marriage and raise children.^129^ The change was subsequently affirmed in the Civil Partnership Act 2004, though elements of the historical approach remain in the sole availability of non-consummation as a ground of nullification for different-sex partners.^130^

In contrast, Lord Millet, although agreeing that the Rent Act violated the Convention rights, argued section 3 HRA 1998 could not be used as it would be too great a departure from the intention behind the legislation. Showing the influence of the historical property focus of family law, he interpreted the Act as not intended to protect loving relationships,^131^ but rather the interest in a property of people who had ‘openly set up home together … as husband and wife’.^132^ As the provision of the Act had its origins in 1920 legislation,^133^ his approach had the support of history.

That Lord Millet’s minority view did not prevail shows the influence of the new powers and different moral basis of human rights upon the conception of relationships within the law. Addressing Lord Millet’s view directly, Lord Rodger argued the Rent Act’s amendment in 1988 to encompass non-married different-sex couples showed the law had already shifted from its historical conception of marriage.^134^ He also pointed to *Fitzpatrick v Stirling Housing Association Ltd*, decided just prior to the HRA 1998’s commencement.^135^ There, without the section 3 HRA 1998 interpretative powers, the Lords were unable to construe the gender-specific words of the Rent Act, ‘as husband and wife’, to include same-sex couples.^136^ However, acknowledging the change the HRA 1998 might bring, they managed to give them protection by interpreting surviving partners as falling within the Act’s wider provision for ‘family’ members to succeed to a tenancy,^137^ encompassing those with a bond of ‘love and affection, not of a casual or transitory nature, but … permanent or at least intended to be so’.^138^

In spite of this promising start, the increased recognition of the modern conception of love under the HRA 1998 has not been smooth. Two years later, in *M v Secretary of State for Work and Pensions*,^139^ the House of Lords refused to find that articles 8 and 14 ECHR were engaged by the failure of the Child Support (Maintenance Assessments and Special Cases) Regulations 1992 to give equal recognition to a same-sex relationship. The regulations required the claimant, whose new partner was the same sex, pay her former partner a greater amount of child support money than if she now lived with a different-sex partner.

The basis of the majority’s judgment was that law and society had only very recently acknowledged that the lack of legal recognition for same-sex relationships was wrong. The regulations were created in 1992, and same-sex relationships were only statutorily recognised two years prior to the judgment in *M*, in the Civil Partnership Act 2004. As a result of the 2004 Act, the law no longer discriminated against M at time of trial, and it was held to be unrealistic for the court to apply

the standards of today to criticise a regime which when it was established represented the accepted values of our society … which, given the size of the overall task and the need to recruit the support of the public, could scarcely have been reformed sooner.^140^

The majority also held the effect of the rules was insufficient to engage the Convention rights protection. Consistent with his decision in *Ghaidan*, Lord Nicholls noted ‘sexual orientation is central to every individual’s personality’.^141^ However, he held the discrimination had not had ‘any significant adverse impact on the claimant’s lifestyle’, and therefore article 8 was not engaged.^142^ Similarly, Lord Walker stated there had been no intrusion into the claimant’s private life: ‘She has not been criminalised, threatened or humiliated.’^143^

At the time, a wide margin of appreciation on the recognition of same-sex relationships was granted by Strasbourg,^144^ and the majority was reluctant early in the HRA 1998’s life to use it to make a bold statement on this point. However, Baroness Hale in a strong dissent argued that the majority’s approach was a step back from *Ghaidan*.^145^ She accepted that it was ‘fair to give the law time to catch up’ and that there was an insufficiently tangible effect on the claimant to amount to a free-standing violation of article 8 or article 1 of Protocol 1.^146^ Nonetheless, she argued that article 8 was engaged and a violation of article 14 should be found.^147^

This back step can be put down to deference and a reluctance to engage in retrospective symbolic judgment. However, the influence of the lack of significant financial harm to the claimant on the majority, in contrast to the facts in *Ghaidan*, also shows vestiges of the law’s view of the importance of the recognition of relationships as a function of the importance of property. It shows a court unwilling to make general use of the HRA 1998 to recognise relationships.

A decade later, *R (Steinfeld and another) v Secretary of State for International Development*^148^ shows the courts moving further towards valuing relationships as manifestations and affirmations of individuals’ identities as choosing beings, consistent with the modern conception of love. Here, a different-sexed couple claimed that the Civil Partnership Act 2004 and Marriage (Same Sex Couples) Act 2013, taken together, violated article 14 in conjunction with article 8 by allowing same-sex couples to choose whether to enter a civil partnership or a marriage, whilst confining different-sex couples to marriage.

Steinfeld and Keidan wanted their relationship legally recognised as a civil partnership because they had ‘ideological objections to marriage based upon … its historically patriarchal nature’.^149^ In the High Court, Mrs Justice Andrews interpreted the decision in *M* as entailing that, as the claim by same-sex partners to legal recognition for their relationship had not fallen within the scope of article 8, neither could a claim by different-sex partners.^150^ Following *M*, she held that for it to be engaged there must be a substantive interference, such as criminalisation or humiliation; mere lack of recognition was insufficient.^151^ She found no evidence that their relationship was devalued as they could obtain the benefits of legal recognition by getting married, and the state had no obligation to make other means of recognition available.^152^

On appeal, both appeal courts found that article 8 was engaged, and the Supreme Court found that article 14 was violated. Lady Justice Arden gave the leading Court of Appeal judgment. In holding article 8 engaged, she relied heavily on the then recent decision in *Oliari v Italy*,^153^ in which the ECtHR narrowed its margin of appreciation and held the failure to offer a means to legally formalise same-sex relationships violated article 8.^154^ Arden LJ held that the ‘positive obligation’ under article 8 to respect family life required the legal recognition of relationships,^155^ as recognition is in itself of value separate from any other effect.^156^ All three members of the Court of Appeal adopted the ECtHR’s reasoning, acknowledging that relationships are an important ‘facet … of an individual’s existence and identity’.^157^ They therefore grounded their judgment, and the importance of recognition, in a conception of relationships consistent with the idea of love as a manifestation and affirmation of individual’s freely chosen identities, influenced by the moral basis of the ECHR.^158^

The understanding of relationships, as intrinsically worthy of recognition and protected under article 8, was not contested by the government when the claimants appealed to the Supreme Court.^159^ The question was whether it, like the Court of Appeal, should show deference to the government’s desire to conduct further research, to ‘wait and see’ if there was a continuing demand for civil partnerships now that same-sex marriage was possible.^160^ Finding that article 14 had been violated, Lord Kerr forthrightly dismissed the government’s arguments.^161^ He held that ‘taking time to evaluate [how to change a discriminatory law] … could never amount to a legitimate aim for the *continuance of the discrimination*’.^162^ The legitimate aim must justify the particular discrimination specifically.^163^ The law was subsequently amended to extend civil partnerships to different-sex couples in the Civil Partnership (Opposite-sex Couples) Regulations 2019.

The extent and the basis of the recognition given to relationships under the Convention rights in *Ghaidan* was called into question in *M*. *Steinfeld* is a reassertion of the modern conception of love, affirming the individual identities of the parties to the relationship by protecting their choice of relationship.

### B. The Limits of Protection of Relationships

The increased willingness to recognise relationships, culminating in *Steinfeld*, involved a conceptual shift in the idea of love recognised by the law. It is consistent with a view of love as an expression and affirmation of the identity of both partners as persons who define their identities through their choices, and is a movement away from love defined by the value of duty or property ownership. This reflects a wider movement in the case law as a result of the influence of the HRA 1998 and ECtHR towards an interpretation of the Convention rights that views and protects individuals as having freedom of choice as to how to live, and away from a view of the individual and their relationship with the state characterised by dutiful obedience.^164^

However, despite this change, I will argue two cases show the protection of the modern account of love is not complete. In *Owens v Owens*^165^ and *R (MM (Lebanon)) v Secretary of State for the Home Department*,^166^ concerns of constitutional competence led the courts to show deference to the other branches of government, demonstrating the continued influence of the values underpinning the historical conception of love within our society and law.

The Supreme Court decision in *Owens* upheld the interpretation of section 1 of the Matrimonial Causes Act 1973, that a lack of a desire to remain together is insufficient grounds for divorce. The Act stated that for divorce, it must be proven ‘the marriage has broken down irretrievably’ using any of five facts showing a breach of a marital duty.^167^ The first three required the claimant prove the respondent is at fault in having committed adultery, other behaviour making it ‘unreasonable to expect the petitioner or applicant to live with’ the respondent^168^ or deserted the claimant for at least two years. The latter two required the parties to have been living apart for two years where the application is not contested by the respondent or for five years where it is.^169^

In this case Mr Owens contested Mrs Owen’s petition for divorce. Not having been living separately for five years, Mrs Owens was compelled to allege ‘the respondent has behaved in such a way that the petitioner cannot reasonably be expected to live with the respondent’.^170^ Both appellate courts held the Central Family Court had correctly applied the law to refuse a divorce.^171^ President Munby upheld the requirement that the applicant prove fault as not contrary to articles 8 and 12 ECHR, noting the ECtHR had held neither article encompassed a right to divorce.^172^ The human rights issue was dropped on appeal to the Supreme Court. Here, the Justices felt serious misgivings about the effect of the limits of the grounds of divorce under the Matrimonial Causes Act in light of the ‘changing social norms … [of] equality between the sexes’.^173^ However, they ruled that as the statute was not unclear, a change to the law to reflect modern values was a matter for Parliament, not the courts.^174^

The courts in *Owens* recognised the inconsistency of the law with modern understandings of relationships as a matter of individual choice. Without the support of the ECtHR and powers of the HRA 1998, they were unable to protect it for reasons of constitutional comity, leaving the claimant ‘trapped in her loveless marriage’ and both parties ‘stymied in lives neither of them wish to lead’.^175^ Although denying the appeal, the Supreme Court gave a clear direction to Parliament that the law should be changed, again demonstrating the role of the courts in leading change to the recognition of the modern conception of relationships. The government subsequently recognised ‘that the law should respect people’s autonomy … at the end of a marriage’ and changed the law in the Divorce, Dissolution and Separation Act 2020.^176^ This Act replaces the requirement to prove the facts of adultery, separation or behaviour making continued cohabitation unreasonable, allowing either or both parties to choose to bring their marriage or civil partnership to an end through an application to the court stating that it has broken down irretrievably.^177^

### C. The Borders of Love

In *Owens*, the courts were faced with a precedent and statute that reflected the values of the past and, without the powers of the HRA 1998, were unable to change it. In the context of immigration laws, the courts have been faced with 21st-century legislation underpinned by the values of dutiful obedience and property, specifically intended to force the separation of partners where one of them is not a UK national, by refusal of leave to remain and deportation or refusal of permission to enter the UK. The courts’ decisions in challenges to these laws demonstrate that whilst they wish to protect the modern conception of love, their ability to do so is ultimately limited by constitutional deference.

In what the government viewed as an excessive number of cases, article 8’s protection of relationships was used to defeat executive decisions to remove individuals from the UK or refuse them leave to remain.^178^ An infamous example is the ‘Catgate’ case, where co-ownership of a cat was part of the evidence that there was a sufficient relationship for the purposes of article 8 to prevent the removal of a non-UK citizen.^179^ In response to these decisions, in 2014 the government inserted sections 117A–D into the Nationality Immigration and Asylum Act 2002,^180^ instructing the courts in their application of the proportionality test under article 8.^181^

Section 117B requires the courts, in considering whether the public interest in allowing removal outweighs the claimant’s article 8 rights, to consider whether ‘it is in the public interest, and in particular in the interests of the economic well-being of the United Kingdom’, that persons seeking to remain in the UK are able to speak English and are financially independent. It adds that ‘little weight’ should be given to ‘private life’ or a relationship formed whilst their immigration status was unlawful or precarious. Under section 117C, the public interest is stated to require the deportation of a ‘foreign criminal’ sentenced to imprisonment for less than four years unless there are significant obstacles to integration in the country to which they would be deported, or its effect on their UK partner or child would be ‘unduly harsh’.

These provisions were intended to make an individual’s choice to create and continue relationships subservient to public interest considerations grounded in the view of the value of the individual defined as obedient—in that they are capable of understanding and obey the laws—and economically productive.^182^ However, the courts have held that whilst sections 117B and 117C require that they must consider the specified public interests, it is the role of the tribunals and courts to determine for themselves on the facts of each case the weight of the claimant’s article 8 interests and whether these are outweighed by those public interests.^183^

The court’s protection of ‘perfectly ordinary family life’ in individual immigration decisions under article 8 has been criticised as ‘trespass[ing] on the executive and legislative functions because they take proportionality so far as to fail to respect sovereignty of Parliament’ by ignoring the intention behind the provisions.^184^ The courts’ restrictive interpretation of section 117B and C is a strong statement that matters of proportionality balancing in individual immigration cases are the courts’ constitutional role, and showed a willingness of the courts to protect the modern choice based conception of love against the values of obedience and property. Government proposals to limit the use of article 8 to prevent deportation show its continued dissatisfaction with this.^185^

The courts’ protection in the immigration context reached its limit in *MM*. Here, the courts were asked to review the compatibility of the minimum income requirement (MIR), which applies to UK citizens seeking to bring their non-UK/EU partners to the UK, with the Convention rights. In contrast to their past recognition and protection of the modern conception of love, perhaps influenced by the wider criticism of the courts’ previous protection of relationships in immigration decisions,^186^ the Supreme Court showed considerable deference. This is inconsistent with the importance of love to human beings and shows the continuing influence of an atavistic duty- and property-based view of relationships within our society.

In order for a UK citizen to bring a partner who is not a UK national (prior to Brexit not a UK or EEA national) to live with them in the UK, the immigration rules require the citizen must earn £18,600, plus an additional £3800 for the first non-UK/EEA child and £2400 for each additional child.^187^ This was challenged under the HRA 1998 using article 8, and the Supreme Court in *MM* recognised that the MIR had ‘caused, and will continue to cause, significant hardship for many thousands of couples … and to their children … [and] may constitute a permanent impediment to many couples’.^188^

Without substantively reviewing the proportionality of the MIR the Supreme Court found that the existence of the MIR did not in itself violate the Convention rights.^189^ The court cited *Konstantinov v Netherlands*, where the ECtHR held rules requiring individuals to ‘demonstrate that he/she has sufficient independent and lasting income, not being welfare benefits’, were not an unreasonable requirement violating article 8.^190^ The ECtHR has repeatedly stated that ‘Article 8 does not entail a general obligation for a State to respect immigrants’ choice of the country of their residence and to authorise family reunion in its territory’.^191^ From this starting point, Lady Hale and Lord Carnwath, giving a joint judgment on behalf of the court, held that the key question was whether the level of the MIR and the Home Office’s discretion to waive it in ‘exceptional circumstances’ was a proportionate interference with article 8.^192^

Formally considering the proportionality test, the court held that the MIR’s ‘aims are no doubt entirely legitimate: to ensure, so far as practicable, that the couple do not have recourse to welfare benefits and have sufficient resources to be able to play a full part in British life’.^193^ The court recognised the rules could mean that some couples could never live together in the UK.^194^ Thirty per cent of UK taxpayers earn less than £18,600.^195^ In applying the proportionality test, the Justices held that deference must be given to the minister’s ‘constitutional responsibility … for setting national policy in this area’ and to ‘the expertise available to her and her department in setting and implementing that policy.’^196^ The court thus held that a MIR was a legitimate and proportionate restriction of the claimant’s article 8 rights.^197^

In reaching this decision, the court did not substantively weigh the proportionality of the MIR, and the Justices did not conduct a balancing exercise and consider the counter arguments that could be made against the legitimate aims relied upon by the Secretary of State. At a practical level, not being allowed into the UK is a greater bar to integration than the lack of money. Additionally, Gemma Manning argues that refusing to allow family unification may result in emotional and financial stress upon an individual, resulting in individuals being less economically productive, more dependent on welfare benefits and less integrated into society.^198^ In legal terms, the law imposes no general obligation to provide welfare benefits; the Convention rights only demand that the state not actively legislate to make an individual destitute,^199^ and the ancient common ‘law of humanity’ sets the low bar that individuals be ‘save[d] … from starving’.^200^ It would not be discrimination contrary to the Equality Act 2010 to instead prohibit non-UK partners from accessing welfare benefits, the position prior to the MIR.^201^

The Supreme Court recognised the love of the partners and the MIR’s interference with it, but in the deference they showed they gave no weight to it.^202^ They held that the aims of the policy were ‘sufficient to justify the interference with, or lack of respect for, the Article 8 right’.^203^ No balancing under the proportionality test occurred, and the claimant’s interests were trumped by the other aims, the weight of which was not assessed or challenged.^204^ In their approach to the existence of the MIR, the court did not balance the desire of UK citizens to live with their partners in the UK against the competing political choices that tax revenue not be spent on non-UK/EEA partners and for integration. However, in line with the decisions on the application of section 117A–D, the court did hold that the application of the MIR in particular cases—those involving children and where an individual has savings or other financial support—could be contrary to article 8.^205^ The rules have now been changed to address this element of the judgment.^206^

In this approach, the court gave deference to the pre-human rights era understanding of love. The choice of the citizen of where to live and who to love are given no weight by the court.^207^ Even Lady Hale, who has been at the forefront of applying the modern conception of love in her judgment in *Ghaidan* and her dissent in *M v Secretary of State for Work and Pensions*, accepted this with apparent sadness but ‘no doubt’.^208^ The measurement of the value to the state of having a citizen within it, and of the citizen being able to choose who to live with in the UK, is defined in economic terms.^209^ In this way, the citizen is treated similarly to a manorial tenant prior to the 14th century who ‘had to pay a “fine” in order to have the lord’s leave to marry’.^210^ We claim to be modern,^211^ but the MIR creates a situation where the citizen, like the serf, is to be forced from their home because of love if they do not have enough money.

The Supreme Court’s finding that the MIR was unlawful insofar as its effect on children confirms the lack of regard for the interests of UK citizens with foreign partners.^212^ The Court held that the application of the immigration rules to separate parents from their children did not comply with the legal requirement to treat ‘the best interests of children as a primary consideration’ in decisions affecting them.^213^ If it is not in the best interests of children to be separated from the parent, it cannot be in the best interests of a person to be separated from their partner. The law expects an adult to choose between two loves: their desire to live within their community in the UK and their partner. The court’s approach implicitly acknowledges that a child, who can feel only the harm that results from either option, cannot adopt the obedient or stoic approach to love the law requires.

This is consistent with the historical conception of love within the law that defined and regulated relationships in terms of duties and property. It also reflects the constitutional position of the citizen under parliamentary sovereignty, preserved by the HRA 1998, as ultimately one defined as a bearer of duties of obedience rather than one with rights to choose how to live.^214^ The only idea of the citizen as a choosing being with which the MIR is consistent is the illusionary one of the citizen as the rational economic actor, free to choose but unprotected from economic pressures.^215^ The citizen is free to choose where to live, but if they cannot meet the MIR they are given the choice of living overseas or without their partner. Although the law has historically recognised the freedom of the citizen to marry who they will, as in past centuries, this is no real choice in the face of ‘enormous physical, moral and economic pressure’ that they must dutifully obey.^216^

Manning ultimately demonstrates the MIR does not even achieve its economic aim of reducing recourse to state funds.^217^ This suggests that the real aim of the policy was to help achieve the government’s previous target of bringing net migration below 100,000;^218^ if a UK citizen must leave the UK to live with their partner, this further reduces net migration. This law is arguably inconsistent with any idea of love. As a reflection of the values of society, of the attitudes of citizens to each other and the state to the citizen, it shows a lack of the desire for togetherness that is common to all the above accounts of love. This attitude can also be seen in the recent stripping of UK citizenship from dual nationals who joined the Islamic State group in Syria.^219^ These laws reflect a relationship between citizens and with the state still influenced by the values of property and obedience.

Individuals are not obliged to feel love for their fellow citizens—to desire togetherness with them—because of the cost to their individual choices such an obligation would impose.^220^ Similarly, the state is not an entity capable of feeling love for its citizens, of desiring togetherness with them.^221^ But Adam Lovett argues that to respect and support the choices of individuals, to the extent that it is not a disproportionate interference with our own or others projects and relationships, is not just to uphold their human rights, but is to act consistently with the modern conception of love.^222^ Respecting the choices of citizens to return to the UK with their partners or to be with their families would be such an act, and consistent with the foundations of human rights.

*MM* marks the limit of the courts’ protection of relationships. In the context of sections 117A–D and under the MIR, the Supreme Court held that it is for the courts to decide in individual cases whether article 8 requires that a relationship be protected. However, in *MM*, they felt constitutionally unable to examine the proportionality of the MIR itself. The deferential decision in *MM*, the MIR and the conception of love implicit within it reflect the values of duty and property that have historically underpinned the idea of love implicit within the law. For the Supreme Court to reach any other outcome would have been a challenge to the other branches of government that was deemed impossible. Yet this is out of step with the choice based recognition of the nature of relationships that the courts have adopted in other areas of the law, where they have not found themselves constrained by the politically controversial nature of the protection of the individual’s choice.

The courts have protected the modern conception of love in their recognition of the existence of relationships as a desire for togetherness in which parties affirm the choice based identity of each other and themselves. The continued presence within society of the duty and property based conception of the individual as an obedient economic subject has caused the courts to show deference, and prevented them from protecting the freedom to choose who to love they have otherwise recognised.

## 5. Conclusion

We have always told love stories, because love is important to us. Although not always engaging with it explicitly, the law has been shaped by understandings of this emotion in a way that reflects the prevailing moral values of society.^223^ The HRA 1998 has required the courts to engage directly with the nature and protection of relationships, and the moral basis of the Convention rights has caused a change to the conception of love they protect: from one defined by duty and property to one that recognises love as a desire for togetherness that affirms the capacity for choice of the lover and the beloved.

Historically, Parliament and the common law’s understanding of love has been shaped by a religious duty based morality and the value of property. As our society’s prevailing morality has changed, so has the law’s understanding of love. Following the creation of the HRA 1998, guided by the ECtHR’s case law, the courts have led the way in advance of legislation, protecting a conception of love underpinned by a morality recognising individuals as persons with the capacity and the right to choose how to live and who to love, reflecting the fundamental basis of the Convention rights.

In the context of the greater recognition of this modern understanding of love, the case of *MM* is anomalous. The deference the court held it was compelled to show allowed the values and conception of love from an earlier age to govern relationships. The judgment left unchallenged views of individuals’ freedom of who to love as limited by their duty to obey the state and pursue financial wealth. The courts give effect to the morality of our society, and in the last 20 years the courts in applying the HRA 1998 have brought considerable change, but our societal morality must change further to truly value the individual’s ability to choose how to live and who to love.

## Footnotes

1 Universal Declaration of Human Rights 1948, preamble, arts 12 and 16; Mary Ann Glendon, *A World Made New* (Random House 2002) 146, 182.

2 Martha C Nussbaum, *Upheavals of Thought: The Intelligence of Emotions* (CUP 2001) 14, 22, 28, 55.

3 James A Brundage, *Medieval Canon Law* (Routledge 1995) 70–3.

4 Mick Jones, *I Want to Know What Love Is* (Atlantic Records 1984).

5 CD Broad, ‘Emotion and Sentiment’ (1954) 13 Journal of Aesthetics and Art Criticism 203, 205; Errol Bedford, ‘Emotions’ (1956–57) 57 Proceedings of the Aristotelian Society 281, 281, 285, 294, 300, 304–5.

6 Cicero, *Tusculan Disputations 406–409* (JE King tr, Harvard UP 1927) 411, 422–3; CS Lewis, *The Four Loves* (William Collins 2012); Nussbaum, *Upheavals of Thought* (n 2) 156.

7 Harry G Frankfurt, *The Reasons of Love* (Princeton UP 2006) 98.

8 Tony Milligan, *Love* (Routledge 2011) 5, 118, 123.

9 Jennifer Terry, ‘Loving Objects’ (2010) 1(2) Trans-Humanities 33, 35–6, 46–50.

10 Milligan (n 8) 5, 118, 133–8.

11 Alasdair Macintyre, *Is Patriotism a Virtue?* (University of Kansas 1984) 4–5.

12 Nussbaum, *Upheavals of Thought* (n 2) 469.

13 ibid 482–3.

14 Plato, *The Symposium* (Penguin 1999) 22–3.

15 ibid 24.

16 Nussbaum, *Upheavals of Thought* (n 2) 565–6, 591–2, 708–9.

17 John Bowlby, *Attachment and Loss*, vol 1 (Hogarth Press 1969) 180–1, 183, 199–202, 209.

18 S Matthew Liao, *The Right to Be Loved* (OUP 2015) 74–99.

19 Erich Fromm, *The Art of Loving* (first published 1956, Harper Collins 1995) 7; Lawrence Friedman and Anke Schreiber, *The Lives of Erich Fromm: Love’s Prophet* (Columbia UP 2013) 173, 182–3; Kieran Durkin, *The Radical Humanism of Erich Fromm* (Palgrave Macmillan 2014) 2–4, 33–4.

20 Fromm, *The Art of Loving* (n 19) 8, 14.

21 Jonathan Herring, *Law and the Relational Self* (CUP 2020) 22–3; Mark Van Hoecke and Mark Warrington, ‘Legal Cultures, Legal Paradigms and Legal Doctrine: Towards a New Model for Comparative Law’ (1998) 47 ICLQ 495, 503.

22 Van Hoecke and Warrington (n 21) 503, 506.

23 ibid 503, 506, 507–8, 511, 536; Drucilla Cornell and Nyoko Muvangua, *uBuntu and the Law: African Ideals and Postapartheid Jurisprudence* (Fordham UP 2012) 2–7.

24 David Pocklington and Frank Cranmer, ‘Banns of Marriage: Their Development and (Possible) Future’ (2017) 19(3) Ecc LJ 342.

25 Universal Declaration of Human Rights 1948, arts 12 and 16; Glendon (n 1); Fareda Banda and John Eekelaar, ‘International Conceptions of the Family’ (2017) 66 ICLQ 833, 835.

26
*Ensslin, Baader and Raspe v Germany* App nos 7572/76, 7586/76 and 7587/76 (ECtHR, 8 July 1978) paras 5, 10.

27
*Schalk and Kopf v Austria* (2011) 53 EHRR 20, para 61; *Oliari v Italy* (2017) 65 EHRR 26, paras 191–2.

28
*Marchx v Belgium* (1979–80) 2 EHRR 14, para 7.

29 Jens M Scherpe, ‘The Right Ambit—Lady Hale and the Limitations of Article 8 ECHR’ (2021) 43 Journal of Social Welfare and Family Law 256, 257–60; *Elsholz v Germany* (2002) 34 EHRR 58, para 43; *Schalk* (n 27) para 91; *Oliari* (n 27) para 173; *Orlandi v Italy* App nos 26431/12, 26742/12, 44057/12 and 60088/12 (ECtHR, 14 December 2017) paras 209–11.

30
*X, Y & Z v UK* (1997) 24 EHRR 143, para 36.

31
*Schalk* (n 27) para 94; *Vallianatos v Greece* (2014) 59 EHRR 12, para 73.

32
*Oliari* (n 27) paras 162, 179–85.

33 UNCHR, ‘Summary of the Human Rights Council Panel Discussion on the Protection of the Family’ (2014) A/HRC/28/40; Banda and Eekelaar (n 25) 835.

34 Text to n 83 below.

35 Nussbaum, *Upheavals of Thought* (n 2) 565–6, 591–2, 708–9.

36 Frankfurt (n 7) 44, 47; Melissa Peskin and Fiona N Newell, ‘Familiarity Breeds Attraction: Effects of Exposure on the Attractiveness of Typical and Distinctive Faces’ (2004) 33 Perception 147.

37 Nussbaum, *Upheavals of Thought* (n 2) 22, 28, 55.

38 ibid 469; Terry A Maroney, ‘Law and Emotion: A Proposed Taxonomy of an Emerging Field’ (2006) 30 Law and Human Behaviour 118, 133.

39 Plato (n 14) 48–9.

40 John Keats, *Selected Poems* (Penguin 2007) 191–2.

41 Plato (n 14) 48.

42 Hannah Arendt, *Love and Saint Augustine* (University of Chicago Press 1996) 25, 96, 562, 565–6, 568, 571; Saint Augustine, *Confessions* (OUP 1998) 140, 150, 182–3, 185; Nussbaum, *Upheavals of Thought* (n 2) 549–50. See also Søren Kierkegaard, *Works of Love* (Harper Collins 2009) 40, 44, 46–7, 49, 52–3.

43 Nussbaum, *Upheavals of Thought* (n 2) 583, 587, 589.

44 Vittorio Montemaggi, *Reading Dante’s Commedia as Theology* (OUP 2016) 32–6, 60–3, 168–71; Dante, *The Divine Comedy* (Clive James tr, Picador 2013) 15.

45 Norman Hampson, *The Enlightenment* (Penguin 1990) 252; Anthony Pagden, *The Enlightenment: And Why It Still Matters* (OUP 2013) 2, 4, 15–16.

46 Nussbaum, *Upheavals of Thought* (n 2) 605–6.

47 Emily Brontë, *Wuthering Heights* (Penguin 2000) 80; see also Nussbaum, *Upheavals of Thought* (n 2) 591, 594–6, 606.

48 Nussbaum, *Upheavals of Thought* (n 2) 604, 607.

49 Brontë (n 47) 80; ibid 608–10.

50 Thank you Rachel Scarfe for pointing this out to me.

51 Charlotte Brontë, *Jane Eyre* (Penguin 2006) 365, 470.

52 Nussbaum, *Upheavals of Thought* (n 2) 711.

53 Nussbaum, *Upheavals of Thought* (n 2) 608–10.

54 Fromm, *The Art of Loving* (n 19) 51, 64–7; Durkin (n 19) 33.

55 Fromm, *The Art of Loving* (n 19) 16.

56 ibid 51–2.

57 ibid 51–2.

58 ibid 15–16.

59 ibid 68; Martin Tucker, ‘The Face of Love’ (1957) 11 Chicago Review 92, 93.

60 Erich Fromm, *To Have or To Be* (Bloomsbury 2013) 21–2.

61 ibid 39; Fromm, *The Art of Loving* (n 19) 19.

62 Fromm, *To Have or To Be* (n 60) 39; cf Tucker (n 59) 93.

63 James Joyce, *Ulysses* (The Modern Library 1992) 333; Nussbaum, *Upheavals of Thought* (n 2) 692–3, 708, 711–12.

64 Joyce (n 63) 733; Nussbaum, *Upheavals of Thought* (n 2) 707, 709.

65 Nussbaum, *Upheavals of Thought* (n 2) 708, 712.

66 Joyce (n 63) 331, 643; Nussbaum, *Upheavals of Thought* (n 2) 708.

67 Joyce (n 63) 732–5, 780; Nussbaum, *Upheavals of Thought* (n 2) 709.

68 Nussbaum, *Upheavals of Thought* (n 2) 707, 709.

69 ibid 478–9, 708–9, 711.

70 Charles Ford, ‘Dante’s Other Brush: Ulysses and the Irish Revolution’ (1992) 28 JJQ 751, 751, 753–4; Patrick Hanafin, *Constituting Identity: Political Identity Formation and the Constitution in Post-independence Ireland* (Dartmouth Publishing 2001) 1–2, 6–8, 10, 99–100, 104–5.

71 Ronald Dworkin, *Is Democracy Possible Here?* (Princeton UP 2008) 9–10, 13, 16–18; Ronald Dworkin, *Justice for Hedgehogs* (Harvard UP 2011) 196–9, 203–5, 209–10, 212, 214.

72 Dworkin, *Is Democracy Possible Here?* (n 71) xi, 7, 32, 35, 45–6; Ronald Dworkin, *Justice for Hedgehogs* (n 71) 204, 337–8.

73 Martha C Nussbaum, ‘Capabilities and Human Rights’ (1997) 66(2) Fordham L Rev 273, 289–90, 292–3, 296; Amartya Sen, ‘Elements of a Theory of Human Rights’ (2004) 32 Philosophy & Public Affairs 315, 334–6.

74 James Griffin, *On Human Rights* (OUP 2008) 33, 35–6, 44–7.

75 Kai Möller, *The Global Model of Constitutional Rights* (OUP 2012) 15–16, 20–1, 30, 57–60, 62–4, 72.

76 Dworkin, *Justice for Hedgehogs* (n 71) 474–5; Möller (n 75) 45, 41, 60.

77 (2002) 35 EHRR 1, para 58.

78 ibid paras 61–2.

79 Nussbaum, *Upheavals of Thought* (n 2) 478–81, 711–12.

80 Robert Alexy, *A Theory of Constitutional Rights* (Julian Rivers tr, OUP 2010) xviii, xxviii, 44, 47–8, 53–4.

81 cf Elizabeth Wicks, ‘The United Kingdom Government’s Perceptions of the European Convention on Human Rights at the Time of Entry’ [2000] PL 438, 444, 447–9.

82 Fromm, *The Art of Loving* (n 19) 16–17.

83 Liao (n 18) 142–50, 202.

84 Council of Europe, *Collected Edition of the Travaux Préparatoires* (Nijhoff 1975) 268; *Johnston v Ireland* (1987) 9 EHRR 203, paras 52–8.

85
*Airey v Ireland* (1979–80) 2 EHRR 305, para 33.

86
*Charalambous v Cyprus* App no 43151/04 (ECtHR, 19 July 2007) para 56; *Ivanov and Petrova v Bulgaria* App no 15001/04 (ECtHR, 14 June 2011) para 61.

87
*Babiarz v Poland* App no 1955/10 (ECtHR, 10 January 2017) para 47.

88 ibid paras 66, 69, 79–80 (Judge Sajó), 99 (Judge Pinto de Albuquerque).

89 Convention on the Elimination of All Forms of Discrimination against Women (1979), arts 1 and 16; UCEDAW, ‘General Recommendation 19’ (1992) UN Doc HRI/GEN/1/Rev.9 (Vol II) paras 11, 22–3; *Shirin Aumeeruddy-Cziffra and 19 Other Mauritian Women v Mauritius* CCPR/C/12/D/35/1978, UN Human Rights Committee (9 April 1981) para 9.2(b)2(ii)1–3; UNCESCR, ‘General Comment 16’ (2005) UN Doc E/C.12/2005/4, para 27; UNCESCR, ‘General Comment 20’ (2009) UN Doc E/C.12/GC/20, para 11; Banda and Eekelaar (n 25) 850–1, 856–7.

90
*Airey* (n 85) paras 13, 33; *Schalk* (n 27) para 94; Jens M Scherpe, *The Present and Future of European Family Law* (Edward Elgar 2016) 2, 5, 27, 57.

91 UN Human Rights Committee, CCPR General Comment No 28: Article 3 (The Equality of Rights Between Men and Women) (29 March 2000) CCPR/C/21/Rev.1/Add.10, para 25.

92 Banda and Eekelaar (n 25) 837, 849–51, 856–7, 859, 861.

93 John Eekelaar, ‘Family Law and Love’ (2016) CFLQ 289, 292–3.

94 ibid 290.

95 Gwynn Davis and Mervyn Murch, *Grounds for Divorce* (Clarendon Press 1988) 31; *Ghaidan v Godin-Mendoza* [2004] UKHL 30, [2004] 2 AC 557 [92]–[94] (Lord Millet).

96 Alan Macfarlane, *Marriage and Love in England, 1300–1840: Modes of Reproduction* (Blackwell 1987) 125.

97 Rebecca Probert, ‘Control over Marriage in England and Wales, 1753–1823: The Clandestine Marriages Act of 1753 in Context’ (2009) 27 LHR 413, 431, 417–18, 431, 433–7, 440, 447–8; Rebecca Probert, *Marriage Law and Practice in the Long Eighteenth Century* (CUP 2009) 210–21.

98 Macfarlane, *Marriage and Love in England* (n 96) 132, 142; Probert, ‘Control over Marriage’ (n 97) 224–5.

99 Nigel Lowe and Gillian Douglas, *Bromley’s Family Law* (11th edn, OUP 2015) 91, citing *The New Jerusalem Bible: Study Edition* (Darton, Longman & Todd 1994) Genesis 2:24, Genesis 3:16.

100 [1982] Ch (CA) 529, 538–9.

101
*Durham v Durham* (1885) 10 PD 80, 82; *Macleod v Macleod* [2008] UKPC 64, [2010] 1 AC 298 [38].

102 Lowe and Douglas (n 99) 92–3.

103 ibid 92–4; *Granatino v Radmacher (Formerly Granatino)* [2010] UKSC 42, [2011] 1 AC 534 [52], [157]. See also *Owens v Owens* [2017] EWCA Civ 182, [2017] 4 WLR 74 [89].

104 Divorce, Dissolution and Separation Act 2020; *Owens v Owens* [2018] UKSC 41, [2018] AC 899.

105 Text to n 176 below.

106
*Balfour v Balfour* [1919] 2 KB 571, 597; Prince Saprai, ‘Balfour v Balfour and the Separation of Contract and Promise’ (2017) 37 LS 468, 470, 473–6, 485–7, 490–1.

107
*Donoghue v Stevenson* [1932] AC 562 (HL) 580; Matthew Chapman, *The Snail and the Ginger Beer: The Singular Case of Donoghue v Stevenson* (Wildy, Simmonds & Hill 2010) 60–1, 110–15; P Applegarth, ‘Lord Atkin: Principle and Progress’ (2016) 90 ALJ 711, 732.

108
*McLoughlin v O’Brian and Others* [1983] 1 AC 410 (HL) 422; *Alcock v Chief Constable of South Yorkshire Police* [1992] 1 AC 310 (HL) 396, 403, 414, 422; *Robertson v Forth Road Bridge Joint Board (No 2)* 1994 SLT (OH) 568, 569, 572.

109 Eekelaar (n 93) 295.

110 Maroney (n 38) 120–1, 133.

111 Eekelaar (n 93) 292.

112 Walter Bagehot, *The English Constitution* (OUP 2001) 190, 192, 199.

113 Brodie Waddell, *God, Duty and Community in English Economic Life 1660–1720* (CUP 2012) 25, 29–35, 45, 78, 87–8, 152–3, 227–30; Enrica Villari, ‘Duty’ (2014) 90 New Left Review 89, 89–90, 95–8; cf Alan Macfarlane, *The Origins of English Individualism* (Blackwell 1994) 165–6, 197.

114 John Curtice, Dominic Abrams and Curtis Jessop, ‘New Values, New Divides?’ in *British Social Attitudes 2020: 38*^*th*^*Report* (The National Centre for Social Research 2020) 19–21.

115
*R (Miller) v Secretary of State for Exiting the European Union* [2016] EWHC 2768 (Admin), [2017] 1 All ER 158 [20]; *R (Miller) v Secretary of State for Exiting the European Union* [2017] UKSC 5, [2018] AC 61 [43].

116 Benedict Douglas, ‘Too Attentive to Our Duty: The Fundamental Conflict Underlying Human Rights Protection in the UK’ (2018) LS 360, 365–7.

117
*R v Secretary of State for the Home Department, ex p Simms* [2000] 2 AC 115, 130, 131; *R (Jackson) v Attorney General* [2005] UKHL 56, [2006] 1 AC 262 [102], [159]; John Laws, ‘The Good Constitution’ (2012) 71 CLJ 567, 581; John Laws, *The Common Law Constitution* (CUP 2014) 43.

118 Tom Bingham, *The Rule of Law* (Penguin 2011) 8; Douglas (n 116) 361, 365, 368, 377–8.

119
*Moschi v Lep Air Services Ltd* [1972] AC 331, 346; Andrew Burrows, *Understanding the Law of Obligations: Essays on Contract, Tort and Restitution* (Hart Publishing 1998) 5–7, 9–10, 14; Stephen Perry, ‘The Role of Duty of Care in a Rights-Based Theory of Negligence Law’ in Andrew Robertson and Hang Wu Tang (eds), *The Goals of Private Law* (Hart Publishing 2009) 81

120 HC Deb 16 February 1998, vol 306, col 781; HL Deb 3 November 1997, vol 582, cols 1227–8, 1308; Paul Boateng and Jack Straw, ‘Bringing Rights Home: Labour’s Plans to Incorporate the European Convention on Human Rights into UK Law’ [1997] EHRLR 71, 80; Francesca Klug, ‘The Human Rights Act 1998, Pepper v Hart and All That’ [1999] PL 246, 247.

121 Joint Committee on Human Rights, *The Case for a Human Rights Commission* (2002–03, HL 67-I, HC 489-I) para 7.

122 Civil Partnership Act 2004; Marriage (Same Sex Couples) Act 2013; Civil Partnership (Opposite-sex Couples) Regulations 2019; Divorce, Dissolution and Separation Act 2020.

123 [2017] UKSC 10, [2017] 1 WLR 771.

124 Rent Act 1977, sch 1, para 2(2).

125
*Ghaidan* (n 95) [17].

126 ibid [35].

127 ibid [142].

128 ibid [132].

129 Eekelaar (n 93) 290, 292; Alexander Maine, ‘The Hierarchy of Marriage and civil partnerships: Diversifying relationship recognition’ in Frances Hamilton and Guido Noto La Diega (eds), *Same-Sex Relationships, Law and Social Change* (Routledge, 2020) 209, 217.

130 Matrimonial Causes Act 1973, s 12(1)(a) and (b); cf Marriage (Same Sex Couples) Act 2013, sch 4, para 4(3); Alexander Maine, ‘Queer(y)ing Consummation: An Empirical Reflection on the Marriage (Same Sex Couples) Act 2013 and the Role of Consummation’ (2021) 33 CFLQ 143, 143–4, 146, 149, 151–3, 155.

131
*Ghaidan* (n 95) [100].

132 ibid [92]–[94].

133 Increase of Rent and Mortgage Interest (Restrictions) Act 1920, s 12(1)(g).

134
*Ghaidan* (n 95) [128].

135 [2001] 1 AC 27.

136 ibid 34, 43, 47, 57.

137 ibid 34, 38–9, 44.

138 ibid 51–2.

139 [2006] UKHL 11, [2006] 2 WLR 637.

140 ibid [6] (Lord Bingham); see also [8] (Lord Nicholls), [91]–[93] (Lord Walker), [149], [151], [153]–[156] (Lord Mance).

141 ibid [32].

142 ibid [32].

143 ibid [88].

144 ibid [96]; *Schalk and Kopf* (n 27) para 105; *Oliari* (n 27) para 163.

145
*M* (n 139) [116], [118].

146 ibid [105], [113]–[115].

147 ibid [111], [118].

148 [2018] UKSC 32, [2020] AC 1.

149
*Steinfeld and another v Secretary of State for Education* [2016] EWHC 128 (Admin), [2016] 4 WLR 41 [5].

150 ibid [31].

151 ibid [28]–[29], [35], [38].

152 ibid [37], [39].

153
*Steinfeld v Secretary of State for Education* [2017] EWCA Civ 81, [2018] QB 519 [25], [27], [62].

154
*Oliari* (n 27) paras 177, 179–84.

155
*Steinfeld* (n 153) [25], [68].

156 ibid [33], [41], [44].

157 ibid [62] (Arden LJ) [137], [141], [143]–[144] (Beatson LJ), [168]–[169] (Briggs LJ).

158
*Oliari* (n 27) para 162.

159
*Steinfeld* (n 149) [19].

160 ibid [27]; *Steinfeld* (n 153) [152], [158], [161]–[162], [164] (Beatson LJ), [170], [175] (Briggs LJ).

161 Andy Hayward, ‘Equal Civil Partnerships, Discrimination and the Indulgence of Time: R (on the application of Steinfeld and Keidan) v Secretary of State for International Development’ (2019) 82 MLR 922, 928.

162
*Steinfeld* (n 149) [46], [50] (emphasis in the original).

163 ibid [42].

164 Douglas (n 116) 371–5, 378.

165
*Owens* (n 104).

166
*MM* (n 123).

167 Matrimonial Causes Act 1973, s 1(1).

168
*Balraj v Balraj* (1981) 11 FLR 110, 112.

169 Matrimonial Causes Act 1973, s 1(2).

170 Matrimonial Causes Act 1973, s 1(2)(b).

171
*Owens* (n 104) [41], [56]; *Owens* (n 103) [45], [53].

172
*Owens* (n 103) [80].

173
*Owens* (n 104) [32], [34]; see generally [36].

174 ibid [45]–[46].

175
*Owens* (n 103) [50], [84], [92]–[98].

176 Ministry of Justice, ‘Reducing Family Conflict: Reform of the Legal Requirements for Divorce’ (September 2019) 25.

177 Divorce, Dissolution and Separation Act 2020, ss 1, 2 and 3.

178 Home Office, Statement of Intent: Family Migration (June 2012) para 7.

179 Asylum and Immigration Tribunal, IA/14578/2008, 24 September 2008; Theresa May, ‘Speech to the 2011 Conservative Party Conference’ (*politics.co.uk*, 4 October 2011) <www.politics.co.uk/comment-analysis/2011/10/04/theresa-may-speech-in-full-2/> accessed 14 April 2021; David Campbell, ‘Catgate and the Challenge to Parliamentary Sovereignty in Immigration Law’ [2015] PL 426, 429–36.

180 Immigration Act 2014, s 19.

181 Campbell (n 179) 427; Joint Committee on Human Rights, *Legislative Scrutiny: Immigration Bill (Second Report)* (2014) paras 109–11.

182 HC Deb 22 October 2013, vol 569, col 162; HL Deb 10 February 2014, vol 752, col 415.

183
*Rhuppiah v Secretary of State for the Home Department* [2018] UKSC 58, [2018] 1 WLR 5536, [49]–[50].

184 Campbell (n 179) 437–9.

185 Ministry of Justice, *Human Rights Act Reform: A Modern Bill of Rights* (CP 588, 2021) 81–2; Bill of Rights Bill (2022–23) 117, cl 8.

186 Ministry of Justice (n 185) 428.

187 Home Office, ‘Immigration Rules Appendix FM: Family Members’, r E-ECP.3.1 (*Immigration Rules*, 25 February 2016) <www.gov.uk/guidance/immigration-rules/immigration-rules-appendix-fm-family-members> accessed 14 December 2020.

188
*MM* (n 123) [80], [81].

189 ibid [41], [58]–[59], [85]–[86].

190 App no 16351/03 (ECtHR, 26 April 2007) paras 48, 50.

191 ibid [48]; *Gül v Switzerland* (1996) 22 EHRR 93, para 38.

192
*MM* (n 123) [41], [44], [61], [68].

193 ibid [82].

194 ibid [80]–[81].

195 HM Revenue and Customs, ‘Percentile Points from 1 to 99 for Total Income Before and After Tax’ (*National Statistics*, 1 December 2012) <www.gov.uk/government/statistics/percentile-points-from-1-to-99-for-total-income-before-and-after-tax> accessed 21 July 2020.

196
*MM* (n 123) [75].

197 ibid [87].

198 Gemma Manning, ‘The Minimum Income Requirement for Family Settlement: The Cost of Integration’ [2019] PL 304, 316–18, 325.

199
*R (Limbuela) v Secretary of State for the Home Department* [2005] UKHL 66, [2005] 3 WLR 1014 [6]–[8], [56]–[57].

200
*R v The Inhabitants of Eastbourne* (1803) 4 East 103; 102 ER 769, 770.

201 Migration Advisory Committee, ‘Review of the Minimum Income Requirement for Sponsorship under the Family Migration Route’ (2011).

202 Michelle Yik Yu Wong, ‘The Unequal Price of Love: A Critical Race Feminist Critique of the £18,600 Minimum Income Requirement for Family Reunification under Article 14 ECHR’ (2019) 33 Journal of Immigration, Asylum and Nationality Law 302, 323–5; Christopher Bertram, Devyani Prabhat and Helena Wray, ‘The UK’s Spousal and Family Visa Regime: Some Reflections after the Supreme Court Judgment in the MM Case’ (*University of Bristol Law School Blog*, 8 March 2017) <https://legalresearch.blogs.bris.ac.uk/2017/03/the-uks-spousal-and-family-visa-regime-some-reflections-after-the-supreme-court-judgment-in-the-mm-case/#more-569> accessed 10 September 2020.

203
*MM* (n 123) [82].

204 Wong (n 202) 302, 315–23.

205
*MM* (n 123) [67]–[68], [91], [100].

206 Home Office, ‘Immigration Rules Appendix FM: Family Members’, rr GEN 3.1, 3.3 and EX 1; Home Office, ‘Immigration Rules Appendix FM-SE: Family Members Specified Evidence’, r 21A (*Immigration Rules*, 25 February 2016) <www.gov.uk/guidance/immigration-rules/immigration-rules-appendix-fm-se-family-members-specified-evidence> accessed 12 August 2020. cf Manning (n 198) 322–3.

207 Bertram, Prabhat and Wray (n 202); Manning (n 198) 316, who notes there is no discussion of citizenship rights by the Supreme Court.

208
*MM* (n 123) [80], [82].

209 Manning (n 198) 317.

210 Macfarlane, *Marriage and Love in England* (n 96) 128.

211 Bruno Latour, *We Have Never Been Modern* (Harvard UP 1993) 26, 28.

212
*MM* (n 123) [92].

213 ibid [89], [91]; Borders, Citizenship and Immigration Act 2009, ss 55(1) and (2).

214 Douglas (n 116) 365–8.

215 Patrick O’Connor, ‘Neo-liberalism and Human Rights’ [2018] EHRLR 541, 549, 555; Manning (n 198) 317.

216 Macfarlane, *Marriage and Love in England* (n 96) 128.

217 Manning (n 198) 317–18, 324.

218 Conservative Party, *Invitation to Join the Government of Britain: The Conservative Manifesto* (2010) 21.

219 Immigration Act 2014, s 66; ‘Shamima Begum Case’ (BBC News, 15 September 2021) <www.bbc.co.uk/news/topics/c347vzp58nzt/shamima-begum-case> accessed 20 December 2021; ‘Jihadi Jack: IS Recruit Jack Letts Loses UK Citizenship’ (BBC News, 18 August 2019) <www.bbc.co.uk/news/uk-49385376> accessed 20 April 2021.

220 J David Velleman, ‘Love as a Moral Emotion’ (1999) 109 Ethics 338, 372; Adam Lovett, ‘The Loving State’ [2022] Inquiry 14–16 <https://doi.org/10.1080/0020174X.2022.2058080> accessed 18 August 2022.

221 Christian List, ‘What Is It Like to Be a Group Agent’ (2016) 52 Noûs 295, 296, 304–6, 311–14; Lovett (n 220) 2–3.

222 Lovett (n 220) 3–4, 14–15, 21, 33.

223 Maroney (n 38) 120–1.

## Notes

I could not have written this article without the advice and encouragement of Eliza Bechtold, Chris Castel, Kirsty Douglas, Ruth Houghton, Theodora Hawksley, Andy Hayward, William Lucy, Roger Masterman, Matthew Nicholson, Aoife O’Donoghue, Mark Ogden, Daniel Orton, Anna Rowlands, Prince Saprai, Gerry Taylor-Aiken, Marion Tait, Irene Wieczorek and the generous anonymous reviewers; I am very grateful. I also benefited from the good-humoured help of those who questioned and commented on it at the SLS and IALT conferences. Any errors are mine.

